# Collision of Epstein–Barr virus-positive and -negative gastric cancer, diagnosed by molecular analysis: a case report

**DOI:** 10.1186/s12876-021-01683-y

**Published:** 2021-03-02

**Authors:** Ken Miyabe, Motonobu Saito, Kei Koyama, Michinobu Umakoshi, Yukinobu Ito, Makoto Yoshida, Yukitsugu Kudo-Asabe, Katsuharu Saito, Hiroshi Nanjo, Daichi Maeda, Keisuke Matsusaka, Akiteru Goto, Koji Kono

**Affiliations:** 1 Department of Cellular and Organ Pathology, Graduate School of Medicine, Akita University, 1-1-1 Hondo, Akita, Akita 010-8543 Japan; 2 Department of Gastrointestinal Tract Surgery, Fukushima Medical University School of Medicine, 1 Hikarigaoka, Fukushima, 960-1295 Japan; 3 Department of Pathology, Akita University Hospital, 1-1-1 Hondo, Akita, Akita 010-8543 Japan; 4 Department of Clinical Genomics, Graduate School of Medicine, Osaka University, 2-2 Yamadaoka, Suita, Osaka 565-0871 Japan; 5 Department of Pathology, Chiba University Hospital, 1-8-1 Inohana, Chuo-ku, Chiba, 260-8677 Japan

**Keywords:** Epstein–Barr virus, Gastric cancer, Collision tumor, Case report

## Abstract

**Background:**

Epstein–Barr virus (EBV)-positive gastric carcinoma (GC) is defined by the proliferation of GC cells with EBV infection. The co-existence of EBV-positive and -negative components in a single GC is rare. We report a case of GC with the co-existence of EBV-positive and EBV-negative components, in which we performed—for the first time—various molecular analyses to elucidate their histogenesis.

**Case presentation:**

An 81-year-old man was diagnosed with GC based on the results of endoscopy and a pathological examination of the biopsy specimen. Systemic chemotherapy was performed, since lymph node and lung metastases were diagnosed based on computed tomography. Total gastrectomy and lymph node dissection were performed after chemotherapy, after confirming that the size of the metastatic lymph nodes had decreased and that the lung metastasis had disappeared. Grossly, a type 3 tumor was located in the middle posterior part of the stomach body. At the cut section, the tumor consisted of a white and solid part on the anal side of the tumor and a flat and elevated part on the oral side. Histologically, the former part consisted of GC with lymphoid stroma and the latter part was composed of poorly differentiated adenocarcinoma without prominent lymphocytic infiltration. The two histopathological components were clearly separated from each other. On EBV-encoded small RNA (EBER)-in situ hybridization (ISH), the part with the lymphoid stroma component was positive, while the other part was negative. Immunohistochemistry revealed that both components showed the overexpression of p53. Sequencing of *TP53* using DNA extracted from the two components was conducted, and revealed different patterns. Targeted next generation sequencing revealed *MYC* amplification in the EBV-positive component of the tumor and *HER2* amplification in the EBV-negative part. Immunohistochemistry revealed that the EBV-positive part was C-MYC( +)/HER2(−) and the EBV-negative part was C-MYC(−)/HER2( +). Correspondingly, chromogenic ISH and dual-color ISH showed amplification of *C-MYC* and no amplification of *HER2* in the EBV-positive part, and no amplification of *C-MYC* and amplification of *HER2* in the EBV-negative part.

**Conclusion:**

We presented a case of collision of two different GCs composed of EBER-ISH ( +)/C-MYC ( +) and EBER-ISH (−)/HER2 ( +) cells.

## Background

Epstein–Barr virus (EBV)-positive gastric carcinoma (GC) is a histological and molecular subtype that is defined by the proliferation of GC cells infected with EBV, demonstrated by EBV-encoded small RNA (EBER)-in situ hybridization (ISH) [1, 2]. EBV-positive GCs account for approximately 10% of GC cases worldwide and have a number of characteristic clinicopathological features, including predominance among males, proximal location in the stomach, lymphoepithelioma-like histology, and a favorable prognosis [1]. Synchronous multiple EBV-positive and -negative GCs are rare, and the co-existence of EBV-positive and -negative components in one GC is rarer [3–5]. To the best of our knowledge, only three cases of gastric cancer with such features have been reported. Among them, only one case of GC with EBV-positive and -negative components was analyzed by p53 immunohistochemistry to investigate its molecular characteristics, which indicated the collision of EBV-positive and -negative components; the other cases were not molecularly analyzed [6–8].

We herein present another case of GC with the co-existence of EBV-positive and -negative components and we performed various molecular analyses (*TP53* sequencing, targeted next generation sequencing, and *HER2* and *C-MYC* ISH) of the EBV-positive and -negative components in order to clarify their histogenesis.

## Case presentation

An 81-year-old Japanese man complained of hematemesis and visited a doctor. He was referred to our hospital with the diagnosis of gastric cancer. Since he had not been seen by a doctor before, there are no special notes in his medical history, including the presence or absence of *H. pylori* infection. Esophagogastroduodenoscopy revealed an ulcer with irregular edges at the middle posterior part of the stomach body. Gastric poorly differentiated adenocarcinoma was diagnosed based on the pathological examination of the biopsy specimen. After making a radiological diagnosis of lymph node and lung metastasis, based on computed tomography findings, the patient was treated with 4 courses of tegafur gimeracil oteracil (S-1) therapy (80 mg/day for 4 weeks with a 2-week rest). After chemotherapy, total gastrectomy and lymph node dissection were performed for the treatment of gastric cancer after confirming that the size of the lymph nodes metastases had decreased and that the lung metastasis had disappeared.

**Fig. 1:**
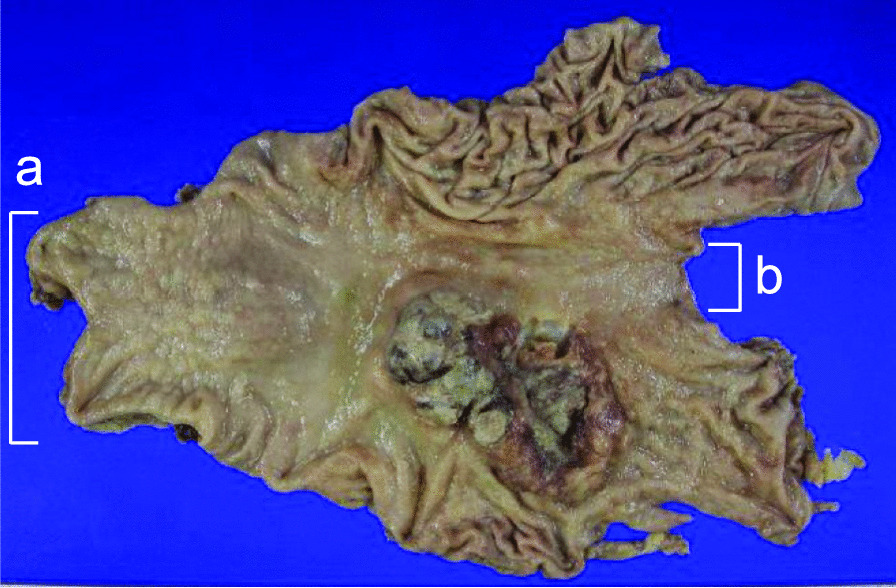
The gross appearance of the gastric tumor. A mucosal view of the total gastrectomy specimen, which was opened along the greater curvature, with the resection margin of the duodenum on the left (**a**) and the gastroesophageal junction on the right (**b**). A type 3 tumor of 83 × 50 mm in size was located in the middle posterior part of the stomach body

**Fig. 2:**
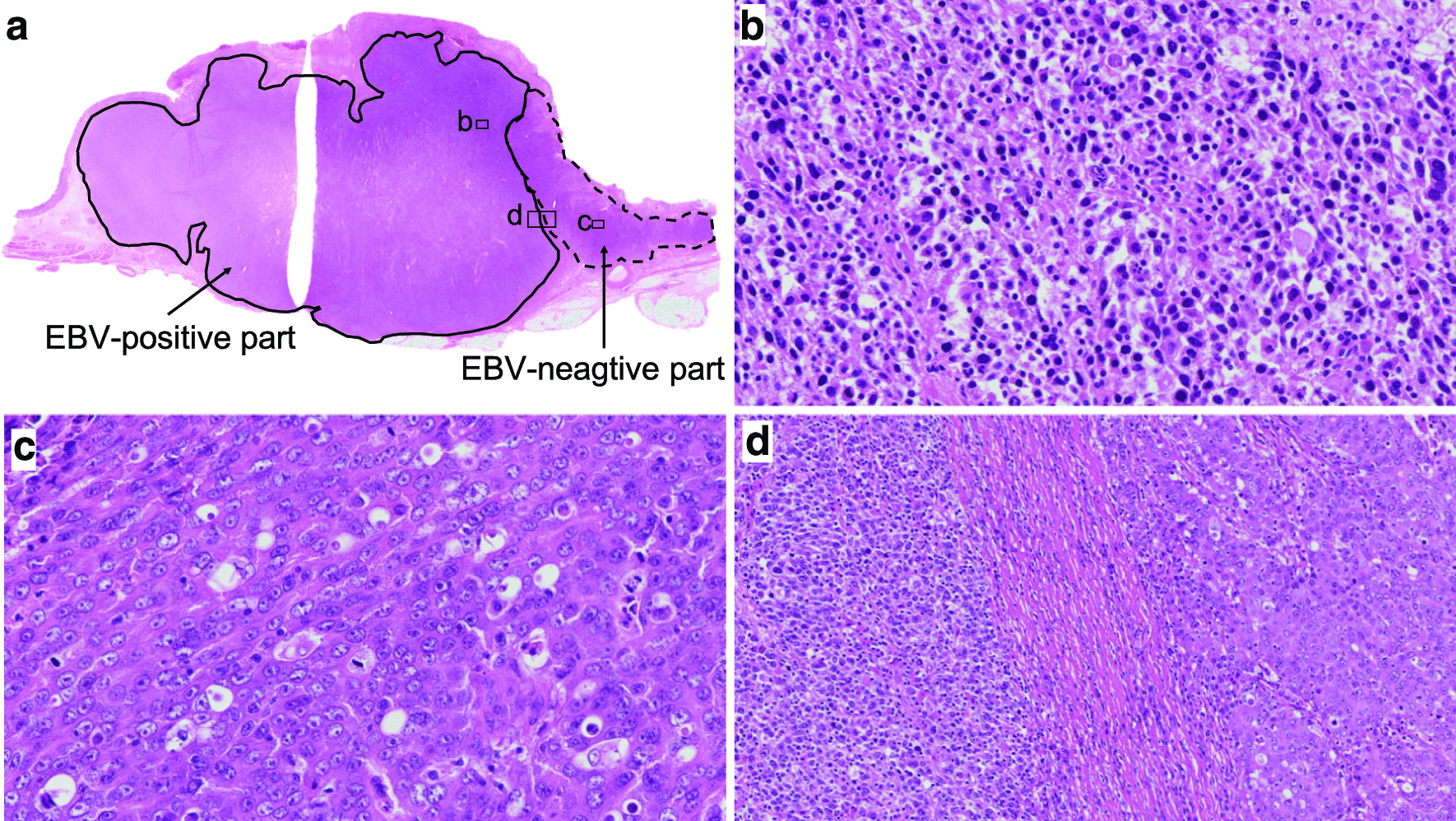
Histological findings of the tumor. **a** Loupe view of the tumor. Hematoxylin and eosin staining (H&E). The EBV-positive and -negative components are outlined by solid and dotted curves, respectively. **b** A high-power view of the EBV-positive part of the tumor, marked with boxed area in **a**. Poorly differentiated adenocarcinoma with dense lymphocytic infiltration, gastric carcinoma with lymphoid stroma was seen (H&E, ×200). **c** A high-power view of the EBV-negative part of the tumor, marked with boxed area in **a**. Poorly differentiated adenocarcinoma without prominent lymphocytic infiltration was seen (H&E, ×200). **d** A low-power view of the border between the two histopathological components marked with boxed area in **a**. The two components were clearly separated from each other (H&E, ×100)

Grossly, a type 3 tumor of 83 × 50 mm in size was located in the middle posterior part of the stomach body (Fig. 1). At the cut section, the tumor consisted of a white and solid part on the anal side of the tumor and flat and an elevated part on the oral side (Fig. 2a).

Histologically, the white and solid part of the tumor consisted of poorly differentiated adenocarcinoma with dense lymphocytic infiltration, GC with lymphoid stroma (Fig. 2b). In contrast, the flat and elevated part was composed of poorly differentiated adenocarcinoma without prominent lymphocytic infiltration (Fig. 2c). These two histopathological components were clearly separated from each other (Fig. 2d). The tumor cells invaded up to the serosa in the gastric carcinoma with lymphoid stroma component. Lymphatic and venous invasion as well as lymph node metastasis were detected, all of which showed gastric carcinoma with lymphoid stroma histology. On EBER-ISH, the GC with lymphoid stroma component was positive, while the other component was negative (Fig. 2a, 3a–c). On EBER-ISH, tumor cells in the metastatic lymph nodes were positive.

**Fig. 3:**
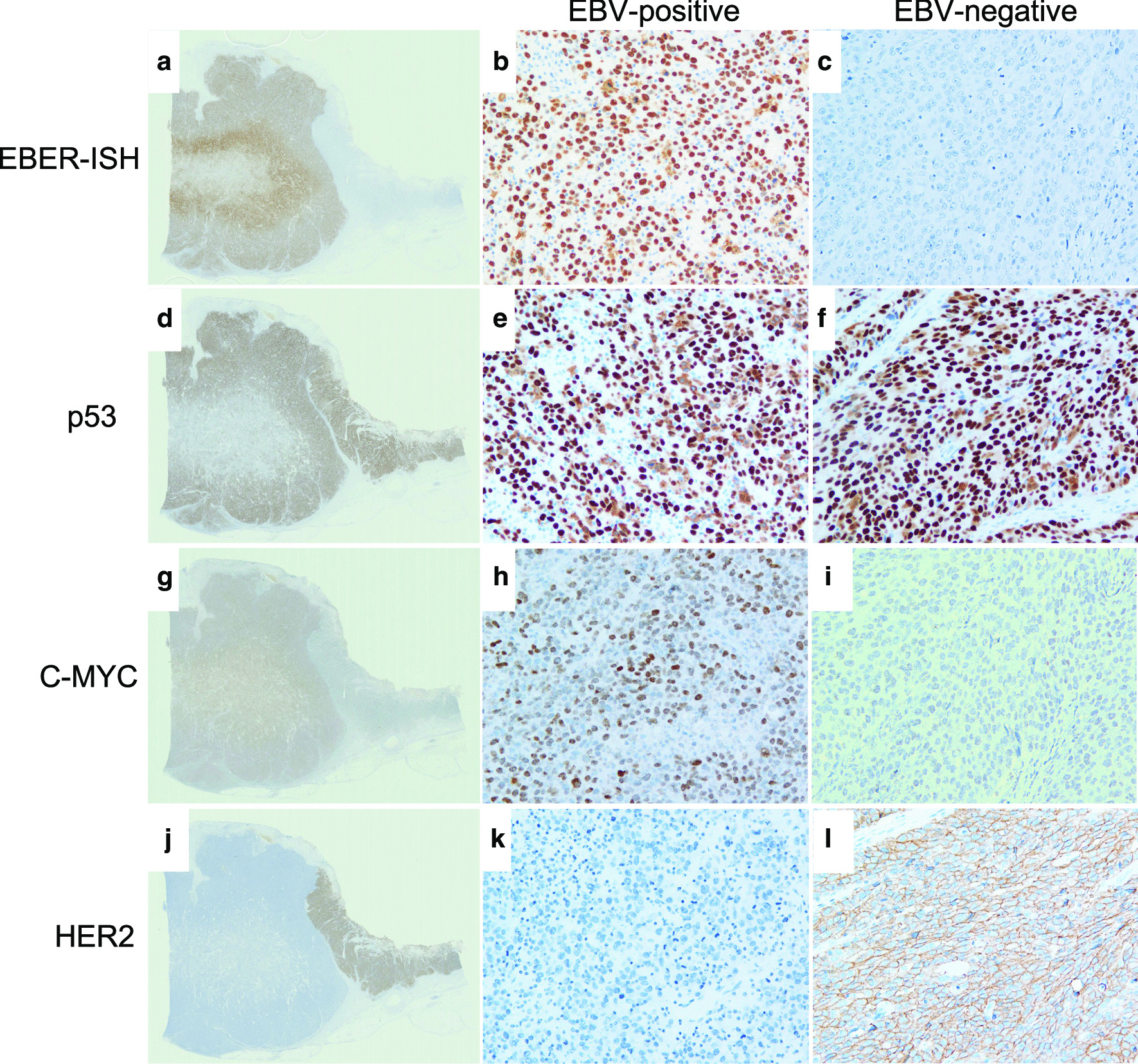
Findings of EBV-encoded small RNA (EBER) in situ hybridization (ISH) and immunohistochemistry. Loupe views of **a** EBER-ISH, **d** p53, **g** C-MYC, and **j** HER2. On EBER-ISH, the gastric carcinoma with a lymphoid stroma component was positive (**b**), while the other component was negative (**c**). Immunohistochemical staining revealed that both the EBV-positive (**e**) and EBV-negative (**f**) components showed the overexpression of p53. The EBV-positive component of the tumor was diffusely positive for C-MYC (**h**), while the EBV-negative component was negative for C-MYC (**i**). The EBV-positive component of the tumor was negative for HER2 (**k**), while the EBV-negative component was positive for HER2 (**l**)

**Fig. 4:**
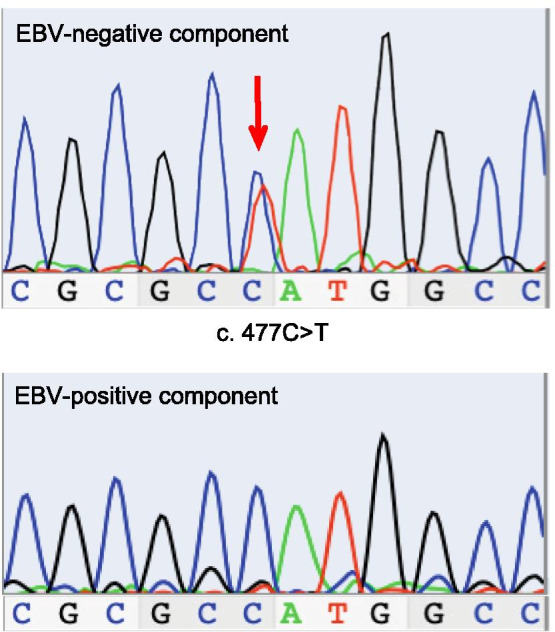
Sequencing chromatograms of *TP53*. The EBV-negative component showed a C→T transition at nucleotide position 477 (c. 477C>T) in exon 5; this transition was not observed at the same nucleotide position of the EBV-positive component

Since both the EBV-positive and -negative components immunohistochemically showed the overexpression of p53 (Fig. 3d–f), exons 5–9 of *TP53* were sequenced using DNA extracted from formalin-fixed paraffin-embedded sections from these two components. The EBV-negative component showed a C→T transition at nucleotide position 477 (c. 477C>T) in exon 5, which gave rise to a synonymous mutation. In contrast, the EBV-positive component showed no mutations at this nucleotide position (Fig. 4). In this study, non-synonymous mutations were not detected in exons 5–9 of *TP53* in either component. These observations are in line with previous results from The Cancer Genome Atlas project, which showed that *TP53* was less frequently mutated in EBV-positive GC [2]. However, it is possible that there may be mutations outside the region that was examined in this study or that mutations could not be detected in DNA extracted from EBV-positive GC because of the large number of lymphocytes and the small percentage of cancer cells.

Targeted next generation sequencing (Oncomine™ Target Test, Thermo Fisher Scientific, Carlsbad, CA), which did not contain a *TP53* test, was performed using DNA and RNA extracted from the EBV-positive component and the EBV-negative component, which revealed *MYC* amplification in the former and *ERBB2* (*HER2*) amplification in the latter.

**Fig. 5:**
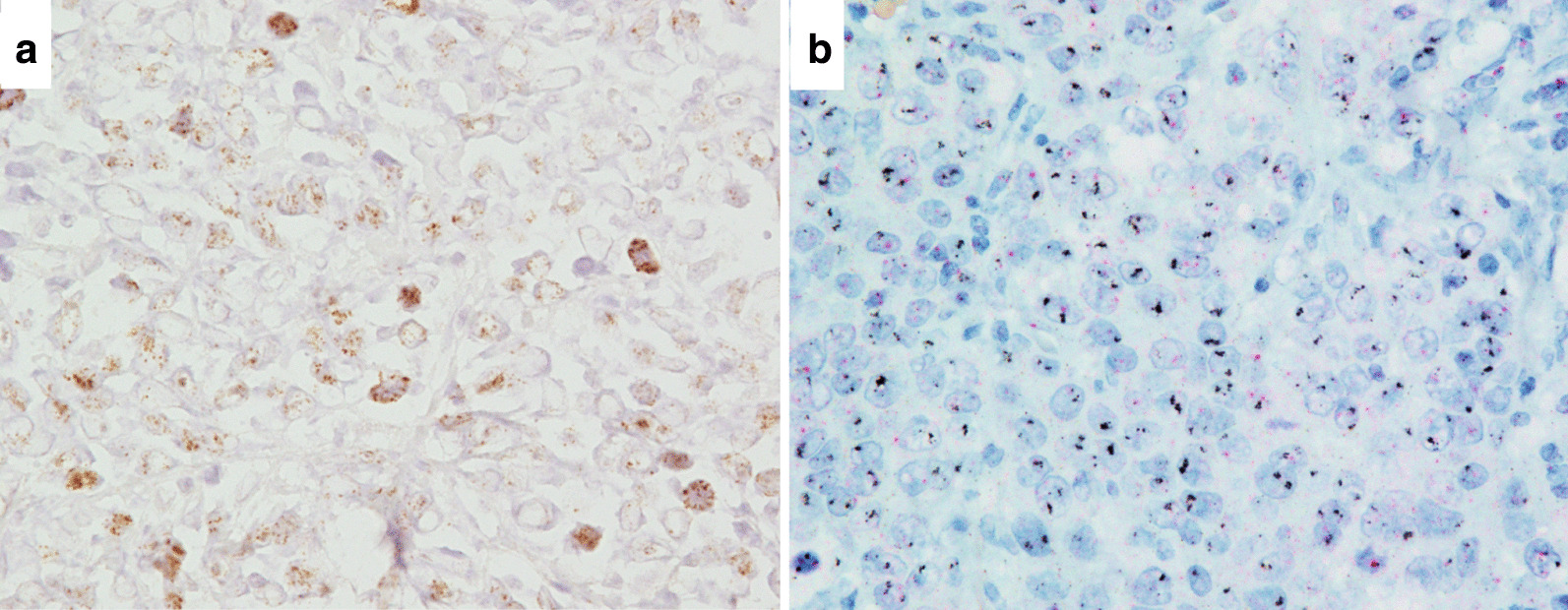
Findings of chromogenic in situ hybridization (ISH). **a** *C-MYC* ISH showed *C-MYC* gene amplification in the EBV-positive part (×400). **b** *HER2* dual-color ISH showed *HER2* gene amplification in the EBV-negative part with a *HER2*/chromosome 17 (Chr17) ratio of 3.9 (×400). Black and red signals represent *HER2* and Chr17, respectively

Immunohistochemically, the EBV-positive component of the tumor was diffusely positive for C-MYC and negative for HER2, while the EBV-negative part was positive for HER2 and negative for C-MYC (Fig. 3g–l). Chromogenic in situ hybridization (CISH) showed high *C-MYC* amplification in the EBV-positive component (Fig. 5a) and no amplification in the EBV-negative part. On dual-color in situ hybridization (DISH) for *HER2*, the *HER2*/chromosome 17 (Chr17) signal count ratio was 3.9 in the EBV-negative component, which was scored as “amplified” (Fig. 5b). In contrast, the *HER2*/Chr17 signal count ratio was 1.4 and the average number of *HER2* signals per cell was 2.8 in the EBV-positive part, which was scored as “not amplified”.

## Discussion and conclusions

The co-existence of EBV-positive and -negative components in one GC is extremely rare; only one case in the relevant English literature and two cases in the relevant Japanese literature have been reported to date [6–8]. Assuming that the histogenesis of such GCs involves the collision of EBV-positive and -negative cancer components, its rarity could be attributed to rarity of co-existing EBV-positive and -negative gastric cancers in a single patient. In fact, in 24 patients with synchronous or metachronous multiple GCs, at least one of which was EBV-positive, the GCs in were all EBV-positive in 15 of 24 cases (62.5%) [3–5, 9, 10]. This percentage is high, considering that EBV-positive GC accounts for only approximately 10% of all GC cases. A high prevalence of EBV-positive GC was reported in synchronous and metachronous multiple gastric cancers and the high frequency would be explained by the acceleration of EBV-associated carcinogenesis by the background mucosa of EBV-positive GC [9, 11].

Regarding the co-existence of EBV-positive and -negative components in a single GC, another possible pathway of histogenesis is the infection or disappearance of EBV in the middle to late steps of GC carcinogenesis. However, this is unlikely, as several studies on EBV-associated gastric carcinogenesis have suggested that EBV infection occurs in the early carcinogenesis of EBV-positive GC, leading to clonal and whole infection in EBV-positive GC [1, 12].

The histological and molecular pathological findings in the present case indicate that it developed via the former pathway, with the nature of the collision demonstrated as follows. (1) The tumor was composed of two histologically different components without any apparent transition between them. (2) The sequencing of exons 5–9 on *TP53* using DNA extracted from the two components showed different patterns. (3) The two components had different patterns of *C-MYC* and *HER2* amplification.

In this case, the EBV-positive component showed the overexpression of C-MYC and the EBV-negative component showed the overexpression of HER2, which corresponded to the gene amplification patterns; however, the causal relationship between these gene amplifications or the overexpression of their proteins and EBV-positive GCs has not been clarified to date [2, 13–16].

*Helicobactor pylori* (*H. pylori*) has long been known to play a major role in gastric carcinogenesis, and the possibility of *H. pylori* involvement should be considered in this case. We therefore subjected a surgical specimen to Giemsa staining and *H. pylori* immunohistochemistry. *H. pylori* infection was not detected in the non-cancerous mucosa of the stomach.

The frequency of nodal metastasis is lower in EBV-positive GCs than in EBV-negative GCs [1, 17, 18]; however, for deeply invasive GCs, the frequency of lymph node metastasis is fairly high, even in EBV-positive GCs [17]. In the present case, the EBV-positive component invaded up to the serosa, while the EBV-negative component invaded up to the subserosa; only the EBV-positive component involved the lymph nodes. It was conceivable that the EBV-positive component, which invaded more deeply than EBV-negative component, metastasized to the lymph nodes.

EBV-positive GCs show a high response rate to immune checkpoint inhibitors [19, 20], and trastuzumab-based chemotherapy is a standard treatment for HER2-positive GC. Thus, in the present case, in which the tumor was composed of EBER-ISH ( +)/HER2 (−) and EBER-ISH (−)/HER2 ( +) components, the response to immune checkpoint inhibitors or trastuzumab-based chemotherapy would be unpredictable. Such unpredictability of the therapeutic effect might be challenge when using molecularly targeted therapy in the treatment of cases with the collision of gastric cancers with different molecular characteristics.

In conclusion, we presented a rare case of collision of two GCs composed of EBER-ISH ( +)/C-MYC ( +) and EBER-ISH (−)/HER2 ( +) cells. This is the first report to describe the analysis of a gastric collision tumor, composed of EBV-positive and -negative components, by targeted next generation sequencing and CISH.

## Data Availability

Additional information is available from the corresponding author on reasonable request from the editor.

## Abbreviations

EBV: Epstein–Barr virus
GC: Gastric carcinoma
EBER: EBV-encoded small RNA
ISH: In situ hybridization
CISH: Chromogenic in situ hybridization
DISH: Dual-color in situ hybridization
Ch17: Chromosome 17
